# Functional Role of the Polymorphic 647 T/C Variant of ENT1 (*SLC29A1*) and Its Association with Alcohol Withdrawal Seizures

**DOI:** 10.1371/journal.pone.0016331

**Published:** 2011-01-24

**Authors:** Jeong-Hyun Kim, Victor M. Karpyak, Joanna M. Biernacka, Hyung Wook Nam, Moonnoh R. Lee, Ulrich W. Preuss, Peter Zill, Gihyun Yoon, Colin Colby, David A. Mrazek, Doo-Sup Choi

**Affiliations:** 1 Department of Molecular Pharmacology and Experimental Therapeutics, Mayo Clinic College of Medicine, Rochester, Minnesota, United States of America; 2 Department of Psychiatry, Mayo Clinic College of Medicine, Rochester, Minnesota, United States of America; 3 Department of Health Sciences Research, Mayo Clinic College of Medicine, Rochester, Minnesota, United States of America; 4 Department of Psychiatry, Psychotherapy and Psychosomatics, Martin-Luther-University, Halle/Saale, Germany; 5 Section Psychiatric Genetics and Neurochemistry, Ludwig-Maximilians University of Munich, Munich, Germany; 6 Department of Psychiatry, University of Minnesota, Minneapolis, Minnesota, United States of America; Tokyo Institute of Psychiatry, Japan

## Abstract

**Background:**

Adenosine is involved in several neurological and behavioral disorders including alcoholism. In cultured cell and animal studies, type 1 equilibrative nucleoside transporter (ENT1, *slc29a1*), which regulates adenosine levels, is known to regulate ethanol sensitivity and preference. Interestingly, in humans, the ENT1 (*SLC29A1*) gene contains a non-synonymous single nucleotide polymorphism (647 T/C; rs45573936) that might be involved in the functional change of ENT1.

**Principal Findings:**

Our functional analysis showed that prolonged ethanol exposure increased adenosine uptake activity of mutant cells (ENT1-216Thr) compared to wild-type (ENT1-216Ile) transfected cells, which might result in reduced extracellular adenosine levels. We found that mice lacking ENT1 displayed increased propensity to ethanol withdrawal seizures compared to wild-type littermates. We further investigated a possible association of the 647C variant with alcoholism and the history of alcohol withdrawal seizures in subjects of European ancestry recruited from two independent sites. Analyses of the combined data set showed an association of the 647C variant and alcohol dependence with withdrawal seizures at the nominally significant level.

**Conclusions:**

Together with the functional data, our findings suggest a potential contribution of a genetic variant of ENT1 to the development of alcoholism with increased risk of alcohol withdrawal-induced seizures in humans.

## Introduction

Alcohol use disorders affect approximately 4–5% of the world population [1], [2] and impose major public health and social problems with substantial worldwide economic loss [3]. For the addicted individuals, the recovery process can be deterred by withdrawal symptoms such as alcohol withdrawal-induced seizures [4]. On a neurochemical level, the withdrawal symptoms are mainly attributed to an imbalance between inhibitory and excitatory neurotransmitters. Alterations of GABA and glutamate levels have been demonstrated to be major contributors of withdrawal symptoms [5], [6]. Recently, several lines of evidence indicated that adenosine promotes GABA release, but inhibits glutamate release in the central nervous system [7]. Consistently, adenosine is known to mediate some aspects of alcohol-induced behavior such as ataxia and hypolocomotion by depressing the neuronal firing activity [8], [9]. Moreover, adenosine exerts anticonvulsant properties through adenosine receptors such as A_1_ or A_2A_ receptors [10], [11], [12]. Several preclinical and clinical trials have suggested that the regulation of adenosine levels might be effective in treating epileptic seizures [13], [14], [15].

Nucleoside transporters regulate adenosine levels across the plasma membrane of neurons and glial cells [7]. *In vitro* studies demonstrated that type 1 equilibrative nucleoside transporter (ENT1) encoded by *Slc29a1* gene is specifically inhibited by acute exposure to alcohol, leading to increased extracellular adenosine levels. However, ENT1 is no longer inhibited by alcohol after chronic alcohol treatment [16], [17], [18]. Our previous studies showed that mice lacking ENT1 are less sensitive to the intoxicating effects of ethanol, and consume more alcohol compared to wild-type littermates [19], [20]. We also found increased glutamate neurotransmission in the striatum as a result of the deficit of presynaptic A_1_ receptor function in the glutamatergic neurons [19] or decreased glutamate uptake activity *via* excitatory amino acid transporter type 2 (EAAT2) in astrocytes [21]. ENT1 is an evolutionarily well-conserved membrane transporter that contains few single nucleotide polymorphisms (SNPs) [22]. After sequencing 200 DNA samples from subjects with European ancestry, Osato et al. found only one nonsynonymous SNP in *ENT1*, 647C (ENT1-216Thr; rs45573936), with a minor allele frequency (MAF) of 0.021 [23]. The function of human ENT1-216Thr in mammalian cells remained unknown. The association between ENT1-216Thr and human psychopathology including alcoholism has also not been studied. Here, we investigated the role of ENT1-216Thr by utilizing three approaches. First, since ENT1 is expressed in most cells, we investigated the pharmacological role of human ENT1-216Thr in primary cultured fibroblast cells derived from ENT1 null mice. Second, we examined ethanol withdrawal seizures in ENT1-null mice compared to wild type littermates. Finally, we tested for association of the ENT1-216Thr variant with alcohol dependence and alcoholism with withdrawal seizures.

## Results

### No Alterations in mRNA and Protein Expressions of ENT1-216Thr

**Figure 1:**
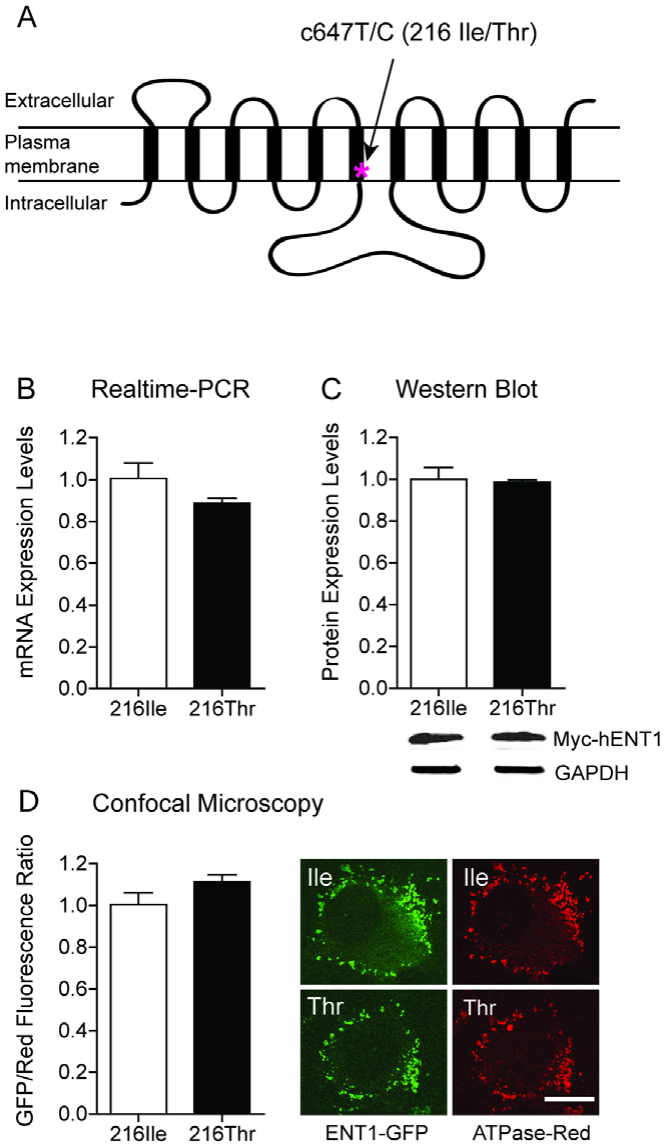
Expression of ENT1-216Thr Variant. (A) A schematic of the transmembrane structure of *hENT1*. The genetic variant of Ile216Thr is located at transmembrane domain 6. Similar mRNA expression (B) and protein expression (C) were found between ENT1-216Ile and 216Thr in transfected MEF cells. (D) Plasma membrane expression of ENT1-216Thr (green) examined by a confocal microscopy. The sodium potassium ATPase (red) was used for maker of plasma membrane. Ratio of green fluorescence versus red one was similar (P>0.05, two-tailed *t*-test). Scale bar = 10 µm. Statistical significance was calculated by two-tailed *t* tests. All data are expressed as mean ± SEM.

Since the 647C (ENT1-216Thr) is the only nonsynonymous SNP (rs45573936) located in transmembrane 6 of human ENT1 (Figure 1A), which possibly alter the ENT1 function [23], we decided to investigate the functional significance of the ENT1-216Thr. We first examined the mRNA and protein expression in mouse embryonic fibroblast (MEF) cells. We utilized MEF cells isolated from ENT1 null mice to eliminate the effects of the endogenous mouse ENT1. The MEF cells were transfected myc-tagged ENT1-216Ile and 216Thr constructs. In a realtime RT-PCR assay, the mRNA levels were similar between ENT1-216Ile and 216Thr (Figure 1B, P>0.05). As shown in Figure 1C, protein levels were also similar between ENT1-216Ile and 216Thr (P>0.05).

Next we examined whether 216Thr alters protein expression in the plasma membrane. Since it was demonstrated that mutations in transmembrane domain 5 [24] and 8 [25] of ENT1 may alter membrane expression of ENT1, we decided to examine whether amino acid changes from isoleucine to threonine in the transmembrane 6 of ENT1 alter membrane expression. Furthermore, the transmembrane 6 is directly linked to a large cytoplasmic domain of the ENT1 which contains several phosphorylation sites. To examine this possibility, we first added enhanced green fluorescence protein (EGFP) to C-terminal part of ENT1-216Ile and 216Thr. We used sodium-potassium ATPase as a plasma membrane marker protein. We, however, found no significant difference of membrane expression between ENT1-216Ile and 216Thr (Figure 1D, P>0.05).

### Prolonged Ethanol Treatment Reduced Binding Affinity, but Increased Uptake Activity of the ENT1-216Thr

**Figure 2:**
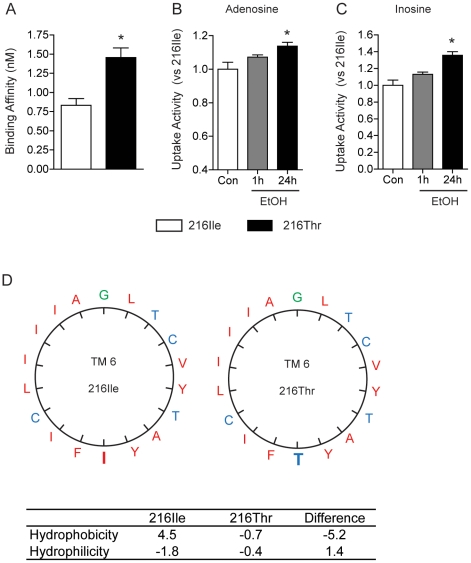
Prolonged Ethanol Exposure Increases Nucleoside Uptake Activity of ENT1-216Thr Compared to ENT1-216Ile. (A) [^3^H] NBMPR bindings demonstrated reduction of K*_d_* in the ENT1-216Ile and -216Thr in MEF cells. (B–C) Prolonged (24 h) ethanol exposure, but not acute exposure (1 h) to ethanol significantly increased the uptakes of adenosine (B) as well as inosine (C) in ENT1-216Thr compared to ENT1-216Ile (*P<0.05, two-tailed *t*-test). All data are expressed as mean ± SEM. (D) Increased hydrophilicity in the helical wheel projection of ENT1-216Thr transmembrane domain 6 in the ExPASy (www.ca.expasy.org). The substitution of 216Ile to 216Thr showed decreased hydrophobicity by 5.2 using Kyte-Doolittle scale (4.5 vs. −0.7) and increased hydrophilicity by 1.4 using Hopp-Woods scale (−1.8 vs. −0.4). Green color represents neutral amino acid residues, red and blue color demonstrates hydrophobic and hydrophilic residues, respectively.

We investigated pharmacological properties of ENT1-216Thr in transfected MEF cells using an ENT1-specific radioligand, [^3^H] NBMPR [S-(*p*-nitrobenzyl)-6-thioinosine] as described [26]. As shown in Figure 2A, we found that the equilibrium dissociation constant (K*_d_*) of ENT1-216Thr (1.46±0.26 nM) was significantly higher compared to ENT1-216Ile (0.83±0.17 nM) (*P<0.05), indicating that the substitution from isoleucine to threonine reduces the binding affinity to NBMPR. On the other hand, the maximal density of transporter sites (B*_max_*) was similar between ENT1-216Ile (4912±225.1 fmol/mg protein) and ENT1-216Thr (5008±213.2 fmol/mg protein) (P>0.05), which is consistent with our Western and confocal microscope analysis in Figure 1. Next, we examined the uptake activity after cells were incubated with 200 mM ethanol for 1 h or 24 h. Interestingly, the uptake activity for both adenosine (Figure 2B) and inosine (Figure 2C) in cells expressing mutant was significantly increased when cells were exposed to ethanol for 24 h while no difference when cells were incubated with ethanol for 1 h.

Since mutation from Ile to Thr could change the hydrophobicity, we examined the hydrophilicity of transmembrane domain 6. This substitution increased hydrophilicity and likely altered the conformation of transmembrane domain 6 (Figure 2D), which might be account for the differential uptake activity in response to prolonged ethanol treatment. We also examined possible compensatory expression of other ENTs. The mRNA expression levels of ENT2, 3, and 4 were similar between ENT1 null mice and wild-type littermates (Figure S1). Thus, our results indicate that the point mutation from isoleucine to threonine in ENT1 increases uptake activity upon prolonged ethanol exposure, which might result in reduced extracellular nucleoside levels including adenosine.

### Increased Ethanol Withdrawal Seizure-like Phenotypes and Decreased Adenosine Levels in ENT1 Null Mice

**Figure 3:**
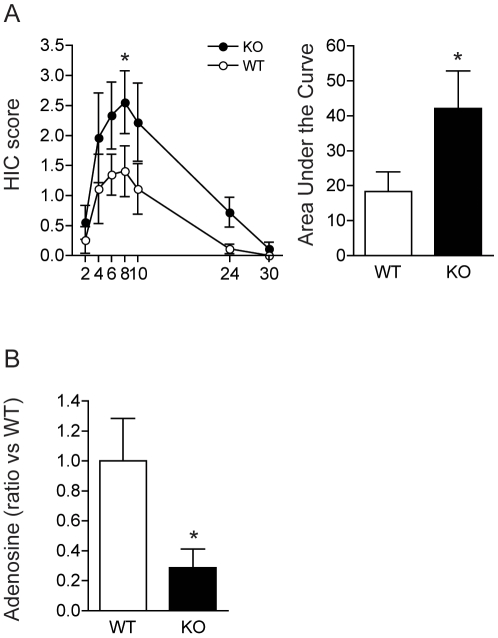
The Absence of ENT1 Increases the Development of Alcohol Withdrawal Seizure in Mice. (A) Handling induced convulsion scores during the 30 h-period following removal from ethanol vapor chambers. ENT1 null mice had higher scores than wild type mice at 8 h after the ethanol withdrawal (*P<0.05, Tukey test). Area under the curve analysis also shows the increased withdrawal seizure in ENT1-null mice compared to wild-type littermates (*P<0.05, two-tailed *t*-test). For each genotype, *n* = 9. (B) Reduced adenosine levels in the striatum of ENT1 null mice compared to wild-type littermates (*P<0.05, two-tailed *t*-test). For each genotype, *n* = 8. All data are expressed as mean ± SEM.

Previously, we found that a deficit of ENT1 in the mouse increases glutamate neurotransmission by decreasing adenosine A_1_ receptor-mediated signaling in the brain [19] or decreased glutamate uptake activity via excitatory amino acid transporter type 2 (EAAT2) in astrocytes [21]. Our recent studies indicate that increased glutamate neurotransmission in the striatum account for some decreased ethanol sensitivity or increased ethanol drinking behaviors in ENT1 null mice [20], [27]. Since glutamate-mediated hyperexcitable state could lead to ethanol withdrawal seizures [6], [28], we investigated whether prolonged ethanol exposure increases withdrawal seizure-like phenotypes using a handling-induced convulsion (HIC) assay for 2–30 h after cessation of ethanol exposure. As shown in Figure 3A, ENT1 null mice showed a significant increased HIC compared to wild-type littermates [F(1,112) = 14.12, P<0.001] and time [F(6,112) = 6.72, P<0.001] effects, without a significant interaction between genotype and time. Further analysis of area under the curve also showed that ENT1 null mice are more susceptible to alcohol withdrawal seizures compared to wild-type mice (Figure 3A). Since striatal adenosine receptors are implicated in ethanol-induced withdrawal seizures [13], we measured total adenosine levels in the striatum using tandem mass spectrometry (Figure 3B). Consistent with our electrophysiology data, we found that adenosine levels were significantly reduced in ENT1 null mice compared to wild-type littermates (P<0.05). These results suggest that deficiency of ENT1 decreases adenosine levels in the striatum, which is implicated in vulnerability to alcohol withdrawal seizures.

### Association of ENT1-216Thr with Alcoholism and the History of Alcohol Withdrawal Seizures

We confirmed the presence of the 647C allele, which encodes the ENT1-216Thr variant by resequencing of PCR-amplificons (Table S1) from 50 alcoholic subjects. The other identified variants were located in non-coding regions, and none of the variants in the intron regions were located in putative sites for alternative splicing (Table S2 and Figure S2). We then genotyped the 647C (Ile216Thr) SNP in 140 additional DNA samples from alcohol-dependent subjects of European descent (total *n* = 190) recruited at the Mayo Clinic in Rochester, Minnesota and 95 DNA samples from self-reported non-alcoholics of European descent available from the Coriell Institute (Camden, NJ). A second sample of 351 alcohol-dependent subjects and 416 non-alcoholic controls from Munich, Germany was also genotyped for this SNP. Clinical characteristics of the study subjects are summarized in Table S3. The ENT1 647T/C (Ile216Thr) SNP was successfully genotyped with a call rate of 100% for Mayo samples and 96% for Munich samples. There were no significant departures from Hardy Weinberg Equilibrium in either sample (P>0.05).

Our functional study in cells with the 216Thr variant of ENT1 protein revealed that prolonged exposure to ethanol resulted in a statistically significant increase in the reuptake of adenosine (Figure 2), which might be a molecular basis of ethanol withdrawal seizures in humans because dysregulation of adenosine levels in the brain is implicated in seizures [13], [14], [15]. Furthermore, we found that mice lacking ENT1 gene, which mimics the hypo-adenosine levels showed increased ethanol withdrawal seizures (Figure 3). Therefore, we investigated the possibility of 647C SNP (216Thr) contributing predisposition to alcoholism characterized by alcohol withdrawal seizures. Among the total of 553 alcohol-dependent subjects included in this study, 79 subjects had a history of alcohol withdrawal seizures (Alc WS) without delirium tremens. Despite the relatively small number of these subjects, focusing on this phenotypically more homogeneous group may increase the power to detect relevant genetic effects.

In the Mayo Clinic sample, the frequency of the ENT1-216Thr variant was twice as high in the group of alcohol-dependent subjects (MAF = 0.032) compared to the Coriell controls (MAF = 0.016), but this difference was not statistically significant (P = 0.27) (Table 1). Comparison between alcoholics with and without a history of seizures showed a trend of association of genotype with seizures [OR = 3.2, 95% CI = (0.8,13.8); P = 0.09] for Mayo subjects. However, there was no significant evidence of association for the Munich subjects (P = 0.70) or the combined group (P = 0.14), although a trend in the same direction was observed [OR = 1.3, 95% CI = (0.3,4.2) in the Munich sample and OR = 1.9, 95% CI = (0.8,4.6) in the combined analysis].

**Table 1:** Association of Ile216Thr Variant with the Phenotypes of Alcohol Dependence in Human Subjects

Site	Groups	Subject (N)	c647 Genotype (N)			Minor Allele	Odds Ratio	Logistic Regression
			TT	TC	CC	Frequency	(95% CI)	P value*
Mayo	Non Alc**	95	92	3	0	0.016	–	–
	Alc-All	190	178	12	0	0.032	2.07 (0.57,7.51)	0.270
	Alc WS***	39	34	5	0	0.064	4.51 (1.02,19.90)	0.047
Munich	Non Alc	416	397	19	0	0.023	–	–
	Alc-All	351	331	20	0	0.028	1.26 (0.66,2.41)	0.48
	Alc WS***	40	37	3	0	0.038	1.69 (0.47,5.99)	0.41
Combined	Non Alc	511	489	22	0	0.022	–	–
	Alc-All	541	509	32	0	0.030	1.41 (0.80,2.47)	0.240
	Alc WS***	79	71	8	0	0.051	2.51 (1.03,6.12)	0.042

*P values indicate the comparison between each subgroup versus non-alcoholic control (Non Alc).

**In Mayo sample, non-alcoholics (Non-Alc) from Coriell Instutitue are self-reported non-alcoholics.

In Munich sample, Non-Alc recruited through an interview as described in the Method section.

***Alc WS denotes that subgroup of alcohol-dependent subjects (Alc-All) with alcohol withdrawal seizures.

Interestingly, in the subgroup of alcohol-dependent subjects with a history of withdrawal seizures (Alc WS), the frequency of the minor allele was approximately four times higher (MAF = 0.064) compared to the non-alcoholic controls (OR = 4.51, 95% CI = (1.02,19.9); P = 0.047). Similar analyses were then performed in the sample of Munich subjects (Table 1). Although no statistically significant associations were observed in this independent sample (P = 0.41), a trend in the same direction was observed, with the rare ENT1-216Thr allele being associated with increased risk of alcoholism with alcohol withdrawal seizures [OR = 1.69, 95% CI = (0.47, 5.99)]. Analysis of the combined Mayo Clinic and Munich samples suggest that the minor ENT1-216Thr allele is more common in alcoholics than in non-alcoholics, and even more common among alcohol dependent subjects with a history of withdrawal seizures, with an odds ratio of 2.51 [95% CI = (1.03, 6.12), P = 0.042] for alcoholism with withdrawal seizures.

## Discussion

This study suggests that a functional variant of human ENT1 gene might contribute to alcoholism with with increased risk of alcohol withdrawal seizures. A recent human study of screening with 1350 variants in 130 genes demonstrated that one genetic variant (rs731780) located in an intron of ENT1 (*SLC29A1*) showed a significant association with heroin addiction in an African-American population [29], attesting for clinical implication of ENT1 in alcohol use disorders and other substance addictions.

**Figure 4:**
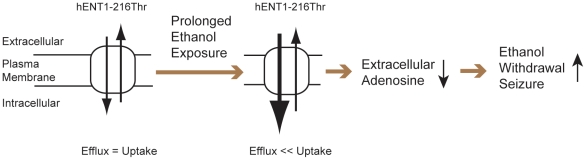
Simplied Schematic Mechanisms of ENT1-Mediated Signaling and Increased Genetic Susceptibility of ENT1-216Thr in Alcohol Withdrawal Seizure. Prolonged ethanol exposure increases uptake activity of ENT1-216Thr compared to ENT1-216Ile, which lead to reduction of extracellular adenosine levels.

Our functional studies using primary cultured cells with the 216Thr variant of ENT1 protein revealed that prolonged exposure to ethanol resulted in a statistically significant increase in the reuptake of adenosine. If the 647 T/C substitution displays the same effect in neuronal cells (increasing uptake activity upon prolonged alcohol exposure), it may cause reduced extracellular adenosine levels and subsequent increase of glutamate tone in the brain, which might be a molecular basis of ethanol withdrawal seizures in humans. Consistently, agonists of both adenosine A_1_ and A_2A_ receptors [13] and NMDA receptor antagonist [28] are known to reduce ethanol-induced withdrawal seizures in mice. Previously, we observed decreased adenosine tones in ENT1-null mice [19], which contribute to decreased levels of ethanol response and excessive alcohol consumption along with increased glutamate neurotransmission in the striatum [19]. Striatal glutamate signaling is known to be involved in ethanol withdrawal seizure-like phenotype [28]. Interestingly, our results indicate that increased uptake function of ENT1-216Thr variant upon alcohol exposure could reduce extracellular or synaptic adenosine levels. Controversially, absence of ENT1 also appears to decrease extracellular adenosine levels in the brain. Since ENT1 is a bidirectional transporter for nucleosides including adenosine and inosine [7], ablation of the ENT1 gene would significantly slow down dynamics of adenosine exchange across the plasma membrane, which may result in low levels of extracellular adenosine and reduced adenosine A_1_ receptor's function in mice [19]. Paradoxically, therefore, increased uptake activity of ENT1 could have a similar consequence as in ENT1 null mice. Moreover, increased alcohol withdrawal seizures in ENT1 null mice extend the phenotypic similarity between ENT1 null mice model and the clinical presentation of alcohol dependence. A possible explanation of the relationships between decreased adenosine neurotransmission, increased alcohol consumption as well as susceptibility to alcohol withdrawal seizure, is presented in Figure 4.

The functional analysis of the ENT1-216Thr in yeast showed no significant differences in basal [^3^H] inosine uptake by nucleoside transporter inhibitors such as ribavirin, cytarabine, and gemcitabine [23]. Although the uptake activities were tested in yeast inactivated endogenous nucleoside transporter, *FUI1*, it has certain limitations to examine the role of mammalian plasma membrane transporter because of different membrane structure, intracellular kinases, and posttranslational modification such as glycosylation which could alter the transporting activity of ENT1. In our study, therefore, we examined the function of ENT1-216Thr in primary cultured fibroblast cells (MEF), which were isolated from ENT1 null mice to rule out possible complications of endogenous ENT1 expression. Moreover, mRNA expression levels of ENT2, 3, and 4, were similar in MEF cells derived from ENT1 null and wild type littermates, indicating that the result was not due to compensatory effects of other ENTs. Also, we found similar plasma membrane expression between ENT1-216Ile and 216Thr in MEF cells, suggesting that membrane trafficking is not the main factor for transporter activity.

The ENT1-216Thr variant exhibited an increased K*_d_* value compared to that of the wild type ENT1-216Ile. This mutation may alter the folding of ENT1, thereby reducing the uptake and binding activity of the transporter. Also, the increased hydrophilicity in transmembrane domains 3 through 6 of ENT1 might alter nucleoside and its analog's transport [24], [25]. The increased hydrophilicity [30] and decreased hydrophobicity [31] values in the helical wheel projection of transmembrane 6 in ENT1 suggest that the substitution from isoleucine to threonine likely changes the conformation of transmembrane domain 6. Subsequently, the threonine residue in the transmembrane domain 6 could alter the third intracellular loop of ENT1, which contains several phosphorylation sites for protein kinase C (PKC), especially PKCδ or ε [32], [33], and casein kinase 2 (CK2) [34]. *In vitro* studies showed a down-regulation of PKCδ or ε decrease ENT1 dependent uptake activity [32]. Interestingly, in cultured neural cells, chronic ethanol exposure increases levels of PKCδ or ε [35], suggesting that increased uptake activity of ENT1-216Thr in response to chronic ethanol exposure, in part, could be mediated by increased PKCδ or ε activity. Since recent findings demonstrated that mice lacking PKCε consume less alcohol and display increased sensitivity to acute intoxicating effects of alcohol [36], [37], while PKCδ null mice show decreased sensitivity to alcohol [38], it is possible that upregulation of PKCε might be responsible for increased uptake activity of ENT1-216Thr compared to that of ENT1-216Ile.

Ethanol induced increased uptake activity both for [^3^H] adenosine and [^3^H] inosine in ENT1-216Thr, which suggests that ethanol may regulate ENT1 function specifically as demonstrated by several *in vitro* studies [17]. Although we observed that an increase of inosine uptake (35.8%) is higher than adenosine uptake (13.8%), this difference might be due to the nucleoside stability since the half-life of adenosine is relatively short (10–30 sec) compared to other nucleosides [39], [40]. In cultured cells, it has been known that acute ethanol exposure increases extracellular adenosine by inhibiting ENT1 uptake activity [17], thereby mediating ataxic or sedative effects of ethanol through adenosine A_1_ and A_2A_ receptors [7]. However, chronic ethanol exposure desensitized the adenosine increase by ENT1 [41], which could contribute to ethanol tolerance. We found that the decreased adenosine activity observed in ENT1 null mice appears to be similar to that of chronic ethanol-induced increase of uptake activity in ENT1-216Thr. Consistently, ENT1 null mice and human alcohol-dependent subjects who carry ENT1-216Thr are able to consume higher amounts of alcohol and are susceptible to ethanol-induced withdrawal seizures (Figure 4). Deletion of the ENT1 gene in mice mimics the state of chronic ethanol exposure, showing decreased levels of ethanol response and excessive alcohol consumption along with decreased synaptic adenosine function and increased glutamate neurotransmission [19], [20].

Alcoholism is a complex multifactorial disease with genetic and environmental components. Identification of contributing factors is challenging, as the effect size of each contribution is likely to be modest. It is expected that focusing on the most severe cases of alcoholism may increase the power to detect genetic effects [42]. Presence of seizures during alcohol withdrawal in mice is widely considered an indicator of severity of alcohol withdrawal and increased glutamate levels [43]. In humans, presence of seizures is also regarded as a sign of severe withdrawal and advanced stage of alcoholism [44], [45], [46]. Thus, selection of this phenotype is reasonable from both genetic and pathophysiological points of view. Consistent with our hypothesis, the 647C variant of the human ENT1 gene was found to be associated with alcoholism with the history of alcohol withdrawal seizures in our first human sample. This same trend was observed in the replication sample but not at a statistically significant level. We performed analysis of the combined data from the two sites using logistic regression with a “site” covariate. We also analyzed the data from the two samples using meta-analysis, which led to similar results with OR (95% CI) = 2.55 (0.97–6.67), P = 0.056.

The human association component of our study is not free from limitations, which include retrospective data collection and reliance on self-report. The retrospective study design precluded us from matching alcoholic study groups on a number of potentially important parameters. Retrospective data collection also poses the risk that important clinical information may be misrepresented. However, among the many possible phenotypes in alcohol dependence, the withdrawal seizure is one of the most obvious and physically noticeable phenotypes for which patients' self-report can be used reliably. Although not perfect, retrospective data collection is commonly used in clinical and genetic studies focused on this phenotype [46]. In an effort to increase the reliability of our data we only included subjects who were able to describe the critically important components of the seizure episode (seizure activity with loss of consciousness) based on information provided by witnesses. Another limitation is the difference between study samples. Subjects recruited in Germany were younger and reported higher alcohol consumption (both on average and maximum per 24 h). In addition, screening for presence of the medical and/or neurological causes for seizures during index episode was performed for subjects recruited in the Mayo sample. These discrepancies between the two study samples might have diminished our ability to detect association between ENT1 647C variant and alcohol withdrawal seizures at a statistically significant level. Nevertheless, combined results from the two independent recruiting sites indicate that the ENT1-216Thr variant may contribute to the risk of alcoholism with withdrawal seizures in humans.

In summary, our studies suggest that ENT1-216Thr appears to contribute to the genetic predisposition to alcoholism with an increased risk of withdrawal-related seizures. Considering the complexity of genetic and environmental contribution to alcohol dependence, this finding is a step towards understanding the genetic predisposition to alcohol-related phenotypes.

## Materials and Methods

### Ethics Statement

This study conducted in compliance with the Code of Ethics of the World Medical Association (Declaration pf Helsinki) and the standard established by the Institutional Review Board of the Mayo Clinic Rochester, Martin-Luther-University and Ludwig-Maximilians University. All subjects provided written informed consent approved by the Institutional Review Board and Ethics Committee of each participating institution. Animal care and handling procedures were approved by Mayo Clinic Institutional Animal Care (approved protocol number: A23808) and Use Committees in accordance with NIH guidelines.

### Mice

ENT1 null mice were generated as described [19]. We used mice having a ∼50% C57BL/6J and ∼50% 129X1/SvJ genetic background to minimize strain-specific effects as recommended [47], [48]. We used 8–16 week old male mice for all experiments. Mice were housed in standard Plexiglas cages with food and water *ad libitum*. The colony room was maintained on a 12 h light/dark cycle with lights on at 6:00 a.m.

### Primary Cell Culture and Transfection

Primary MEF cells were prepared from ENT1 null mice at embryonic day 13.5 postcoitum. MEF cells were grown in DMEM supplemented with 10% heat-inactivated fetal bovine serum and 100 µg/ml penicillin/streptomycin at 37°C in a water-saturated atmosphere under 5% CO_2_ in air. Cells were transfected using Lipofectamine and PLUS reagents (Invitrogen, Carlsbad, CA).

### Site-directed Mutagenesis

Human cDNA of ENT1, which is cloned into pcDNA3, was provided kindly by Dr. Pastor-Anglada M. (University of Barcelona, Spain). A point mutation was generated using QuikChange site-directed mutagenesis kit (Stratagene, La Jolla, CA). The cDNAs of 216Ile wild type and 216Thr variant were subcloned into pCMV-Myc (Clontech, Mountain View, CA) and pEGFP-N1 (Clontech) respectively.

### Expression Measurements

After transfection, 1 µg of RNA was converted to cDNA by reverse transcription reaction using Omniscript (Qiagen, Valencia, CA) and random hexamer primers. Realtime PCR was performed by IQ SYBR Green Supermix (Bio-Rad, Hercules, CA) using MyiQ realtime PCR detection system (Bio-Rad). Relative mRNA expressions, which were normalized by beta-actin were calculated by using the 2^−ΔΔCt^ method [49]. We used anti-myc antibodies to examine hENT1 protein since no specific antibodies are available for mouse or human ENT1. To determine the protein expression by Western blot, proteins prepared from transfected cells were separated on 4–12% Bis-Tris gels. Proteins in gels were transferred to nitrocellulose membranes, which were incubated for 2 h at 4°C in blocking buffer containing 5% BSA and 0.05% Tween 20 in Tris-buffered saline (10 mM Tris, pH 7.5, and 100 mM NaCl). The membranes were incubated overnight at 4°C with c-Myc antibody (1∶1,000, Clontech, supplied with c-Myc vector) and then re-probed with GAPDH antibody (1∶300, Chemicon, Temecula, CA) to assess the protein amount.

### Fluorescence Microscopy

MEF cells were transfected and grown on coverglasses and then fixed in 2% paraformaldehyde for 30 min at room temperature. For plasma membrane marker, sodium potassium ATPase was used. After blocking in 5% goat serum, the slides were incubated for 1 h at room temperature with primary sodium potassium ATPase antibody for plasma membrane marker (1∶100, Abcam, Cambridge, MA) and secondary Rhodamine Red™-X goat anti–mouse IgG antibody (1∶100, Invitrogen). Fluorescence was analyzed using a LSM510 confocal microscope (Carl Zeiss, Germany). The green and red fluorescence were quantified using Image J (NIH).

### Uptake Assays

MEF cells were transfected with each construct of *hENT1*-216Thr and 216Ile. The [^3^H] nucleoside uptake assay was conducted in phosphate-buffered saline (PBS) containing 1 µCi [^3^H] nucleoside/ml at room temperature for 30 min in the presence or absence of ENT1-specific inhibitor (NBMPR). Uptake was terminated by adding ice-cold PBS containing 10 µM NBMPR and cells were then solubilized in 1% Triton X-100. An aliquot was taken for measurement of radioactivity and protein content using, respectively, a liquid scintillation counting and protein assay.

### [^3^H] NBMPR Binding

The transfected MEF cells were incubated with [^3^H] NBMPR with and without competitive ENT1-specific inhibitor dilazep (10 µM) for 45 min at room temperature. Cells were washed with cold Tris-HCl (10 mM, pH 7.4), and then solubilized in 1% Triton X-100. An aliquot was taken for measurement of radioactivity and protein content using, respectively, a liquid scintillation counting and protein assay. Specific binding was defined as total minus nonspecific binding. The K*_d_* and B*_max_* values were calculated from nonlinear curve fits (Prism 4, GraphPad Software, San Diego, CA).

### Handling-Induced Convulsion (HIC) Score

To examine whether absence of ENT1 alters ethanol withdrawal seizures, we measured handling induced convulsions (HIC) scored up to 30 h after 3 d (16 h/day) of ethanol exposure in vapor chamber (7–10 mg/L of air, 150∼200 mg/dl blood ethanol levels) as described [50].

### Adenosine Levels

We measured striatal adenosine levels using a modified LC/MS/MS (liquid chromatography and tendem mass spectrometry) method as described [51]. Briefly, striatal tissues were heated to 70°C for 20 min followed by addition of ice-cold 10 mM HCl and centrifuged to precipitate proteins at 25,000 *g* for 10 min at 4°C. Followed by, supernatants were desalted using Oasis HLB sample cartridges (Waters, Malford, MA) and then each sample was diluted (200 ng/µl protein) and stored at −20°C until use. 2 µg samples (10 µl) were analyzed using Paradigm MS4B HPLC (Michrom Bioresources, Auburn, CA) coupled with a TSQ Quantum Ultima MS triple quadrupole mass spectrometer (Thermo Fisher Scientific, Waltham, MA). Separation was performed with Atlantis dC18 NanoEase Column (3 µm, 300 µm×100 mm) with 5 µl/min flow rate. Using Multiple Reaction Monitoring (MRM) scans, adenosine levels were quantified by measuring dissociation of adenosine (268 Da) to fragmented compound (136 Da) in 20 eV collision energy.

### Human Subjects, DNA Sequencing and Genotyping

Human subjects were recruited from Mayo Clinic Rochester and Munich, Germany (see detailed description in the Methods S1). To confirm genetic variation in human ENT1, new pairs of primers were generated for all exons of the human ENT1 gene (Table S1), including about 100 bp of exon-intron boundaries based on the chromosome 6p21.1 genomic sequence (NT_007592.15). Genomic DNA was isolated from blood of patients using AutoPure LS (Gentra, Minneapolis, MN). The coding sequence of the human ENT1 was compared with the GenBank accession number NM_004955. All the exons and introns were PCR amplified and sequenced using ABI 3730xl automated sequencer (Applied Biosystems). Sequence variants were then screened by Mutation Surveyor version 2.2 (Softgenetics, PA). For 647C (ENT1-216Thr) genotyping, TaqMan assay was employed using the ABI prism 7900HT sequence detection system (Applied Biosystems, Foster City, CA).

### Statistics

Data are presented as mean ± SEM (standard error of the mean). Data were examined by two-tailed *t*-test or two-way ANOVA (ethanol-induced withdrawal seizure) followed by a Tukey *post-hoc* test for comparison among different treatment groups. For statistical analysis for human genomics, see the Methods S1. Results were considered significantly different when P<0.05.

## Supporting Information

Methods S1 **Study Subjects and Statistics.**
(DOC)

Figure S1 **mRNA expression of ENT2, 3, and 4 in ENT1-null MEF cells.**
(TIF)

Figure S2 **Schematic representation of human ENT1 gene and variants discovered from alcoholics in this study.**
(TIF)

Table S1 **List of Primers for Genomic DNA Fragment Amplication for Resequencing.**
(DOC)

Table S2 **Genetic Variants of ENT1 (*SLC39A1*) in Alcohol-Dependent Subjects.**
(DOC)

Table S3 **Summary of Clinical and Demographical Information of Subjects.**
(DOC)

## References

[1] Ezzati M, Lopez AD, Rodgers A, Vander Hoorn S, Murray CJ (2002). Selected major risk factors and global and regional burden of disease.. Lancet.

[2] Robins LN, Helzer JE, Weissman MM, Orvaschel H, Gruenberg E (1984). Lifetime prevalence of specific psychiatric disorders in three sites.. Arch Gen Psychiatry.

[3] Harwood HJ, Fountain D, Livermore G (1998). Economic costs of alcohol abuse and alcoholism.. Recent Dev Alcohol.

[4] Choi D-S, Karpyak VM, Frye MA, Hal-Flavin DK, Mrazek DA, Waldman SA, Terzic A (2009). Drug Addiction, in Pharmacology and Therapeutics: Principles to Practice.

[5] Liskow BI, Goodwin DW (1987). Pharmacological treatment of alcohol intoxication, withdrawal and dependence: a critical review.. J Stud Alcohol.

[6] Tsai G, Coyle JT (1998). The role of glutamatergic neurotransmission in the pathophysiology of alcoholism.. Annu Rev Med.

[7] Dunwiddie TV, Masino SA (2001). The role and regulation of adenosine in the central nervous system.. Annu Rev Neurosci.

[8] Dar MS (1990). Central adenosinergic system involvement in ethanol-induced motor incoordination in mice.. J Pharmacol Exp Therapeutics.

[9] Dar MS (1990). Functional correlation between subclasses of brain adenosine receptor affinities and ethanol-induced motor incoordination in mice.. Pharmacol Biochem Behav.

[10] Boison D (2005). Adenosine and epilepsy: from therapeutic rationale to new therapeutic strategies.. Neuroscientist.

[11] Boison D (2006). Adenosine kinase, epilepsy and stroke: mechanisms and therapies.. Trends Pharmacol Sci.

[12] Dragunow M (1991). Adenosine and seizure termination.. Ann Neurol.

[13] Kaplan GB, Bharmal NH, Leite-Morris KA, Adams WR (1999). Role of adenosine A1 and A2A receptors in the alcohol withdrawal syndrome.. Alcohol.

[14] Boison D (2008). Adenosine as a neuromodulator in neurological diseases.. Curr Opin Pharmacol.

[15] Jacobson KA, Gao ZG (2006). Adenosine receptors as therapeutic targets.. Nat Rev Drug Discov.

[16] Gordon AS, Collier K, Diamond I (1986). Ethanol regulation of adenosine receptor-stimulated cAMP levels in a clonal neural cell line: An in vitro model of cellular tolerance to ethanol.. Proc Natl Acad Sci USA.

[17] Nagy LE, Diamond I, Casso DJ, Franklin C, Gordon AS (1990). Ethanol increases extracellular adenosine by inhibiting adenosine uptake via the nucleoside transporter.. J Biol Chem.

[18] Sapru MK, Diamond I, Gordon AS (1994). Adenosine receptors mediate cellular adaptation to ethanol in NG108-15 cells.. J Pharmacol Exp Therapeutics.

[19] Choi DS, Cascini MG, Mailliard W, Young H, Paredes P (2004). The type 1 equilibrative nucleoside transporter regulates ethanol intoxication and preference.. Nat Neurosci.

[20] Chen J, Nam HW, Lee MR, Hinton DJ, Choi S (2010). Altered glutamatergic neurotransmission in the striatum regulates ethanol sensitivity and intake in mice lacking ENT1.. Behav Brain Res.

[21] Wu J, Lee MR, Choi S, Kim T, Choi D-S (2010). ENT1 regulates ethanol-sensitive EAAT2 expression and function in astrocytes.. Alcohol Clin Exp Res.

[22] Leabman MK, Huang CC, DeYoung J, Carlson EJ, Taylor TR (2003). Natural variation in human membrane transporter genes reveals evolutionary and functional constraints.. Proc Natl Acad Sci U S A.

[23] Osato DH, Huang CC, Kawamoto M, Johns SJ, Stryke D (2003). Functional characterization in yeast of genetic variants in the human equilibrative nucleoside transporter, ENT1.. Pharmacogenetics.

[24] SenGupta DJ, Lum PY, Lai Y, Shubochkina E, Bakken AH (2002). A single glycine mutation in the equilibrative nucleoside transporter gene, hENT1, alters nucleoside transport activity and sensitivity to nitrobenzylthioinosine.. Biochemistry.

[25] Visser F, Sun L, Damaraju V, Tackaberry T, Peng Y (2007). Residues 334 and 338 in transmembrane segment 8 of human equilibrative nucleoside transporter 1 are important determinants of inhibitor sensitivity, protein folding, and catalytic turnover.. J Biol Chem.

[26] Hammond JR (2000). Interaction of a series of draflazine analogues with equilibrative nucleoside transporters: species differences and transporter subtype selectivity.. Naunyn-Sch Arch Pharmacology.

[27] Nam HW, Lee MR, Hinton DJ, Choi DS (2010). Reduced effect of NMDA glutamate receptor antagonist on ethanol-induced ataxia and striatal glutamate levels in mice lacking ENT1.. Neurosci Lett.

[28] Rossetti ZL, Carboni S (1995). Ethanol withdrawal is associated with increased extracellular glutamate in the rat striatum.. Eur J Pharmacol.

[29] Levran O, Londono D, O'Hara K, Randesi M, Rotrosen J (2009). Heroin addiction in African Americans: a hypothesis-driven association study.. Genes Brain Behav.

[30] Hopp TP, Woods KR (1981). Prediction of protein antigenic determinants from amino acid sequences.. Proc Natl Acad Sci U S A.

[31] Kyte J, Doolittle RF (1982). A simple method for displaying the hydropathic character of a protein.. J Mol Biol.

[32] Coe I, Zhang Y, McKenzie T, Naydenova Z (2002). PKC regulation of the human equilibrative nucleoside transporter, hENT1.. FEBS Lett.

[33] Coe IR, Dohrman DP, Constantinescu A, Diamond I, Gordon AS (1996). Activation of cyclic AMP-dependent protein kinase reverses tolerance of a nucleoside transporter to ethanol.. J Pharmacol Exp Therapeutics.

[34] Bone DB, Robillard KR, Stolk M, Hammond JR (2007). Differential regulation of mouse equilibrative nucleoside transporter 1 (mENT1) splice variants by protein kinase CK2.. Mol Membr Biol.

[35] Messing RO, Petersen PJ, Henrich CJ (1991). Chronic ethanol exposure increases levels of protein kinase C d and e and protein kinase C-mediated phosphorylation in cultured neural cells.. J Biol Chem.

[36] Choi DS, Wang D, Dadgar J, Chang WS, Messing RO (2002). Conditional rescue of protein kinase C epsilon regulates ethanol preference and hypnotic sensitivity in adult mice.. J Neurosci.

[37] Hodge CW, Mehmert KK, Kelley SP, McMahon T, Haywood A (1999). Supersensitivity to allosteric GABA(A) receptor modulators and alcohol in mice lacking PKCepsilon.. Nat Neurosci.

[38] Choi DS, Wei W, Deitchman JK, Kharazia VN, Lesscher HM (2008). Protein kinase Cdelta regulates ethanol intoxication and enhancement of GABA-stimulated tonic current.. J Neurosci.

[39] Ballarin M, Fredholm BB, Ambrosio S, Mahy N (1991). Extracellular levels of adenosine and its metabolites in the striatum of awake rats: inhibition of uptake and metabolism.. Acta Physiol Scand.

[40] Nagel J, Hauber W (2002). Effects of salient environmental stimuli on extracellular adenosine levels in the rat nucleus accumbens measured by in vivo microdialysis.. Behav Brain Res.

[41] Nagy LE, Diamond I, Collier K, Lopez L, Ullman B (1989). Adenosine is required for ethanol-induced heterologous desensitization.. Mol Pharmacol.

[42] Schork NJ, Schork CM (1998). Issues and strategies in the genetic analysis of alcoholism and related addictive behaviors.. Alcohol.

[43] Tsai GE, Ragan P, Chang R, Chen S, Linnoila VM (1998). Increased glutamatergic neurotransmission and oxidative stress after alcohol withdrawal.. Am J Psychiatry.

[44] Duka T, Gentry J, Malcolm R, Ripley TL, Borlikova G (2004). Consequences of multiple withdrawals from alcohol.. Alcohol Clin Exp Res.

[45] Schmidt LG, Sander T (2000). Genetics of alcohol withdrawal.. Eur Psychiatry.

[46] Schuckit MA, Tipp JE, Reich T, Hesselbrock VM, Bucholz KK (1995). The histories of withdrawal convulsions and delirium tremens in 1648 alcohol dependent subjects.. Addiction.

[47] Banbury Conference on Genetic Background in Mice (1997). Mutant Mice and Neuroscience: Recommendations Concerning Genetic Background.. Neuron.

[48] Crusio WE, Goldowitz D, Holmes A, Wolfer D (2009). Standards for the publication of mouse mutant studies.. Genes Brain Behav.

[49] Livak KJ, Schmittgen TD (2001). Analysis of relative gene expression data using real-time quantitative PCR and the 2(-Delta Delta C(T)) Method.. Methods.

[50] Becker HC, Diaz-Granados JL, Hale RL (1997). Exacerbation of ethanol withdrawal seizures in mice with a history of multiple withdrawal experience.. Pharmacology Biochemistry & Behavior.

[51] Che FY, Zhang X, Berezniuk I, Callaway M, Lim J (2007). Optimization of neuropeptide extraction from the mouse hypothalamus.. J Proteome Res.

