# Supplementary material for: Functional Role of the Polymorphic 647 T/C Variant of ENT1 (SLC29A1) and Its Association with Alcohol Withdrawal Seizures
Source: PLoS One. 2011 Jan 24;6(1):e16331. doi: 10.1371/journal.pone.0016331 (PMC3026043; doi:10.1371/journal.pone.0016331)
Supplement: Methods S1 — Study Subjects and Statistics. (DOC) [file pone.0016331.s001.doc]

**Methods S1**

**Study Subjects.** For the Mayo Clinic sample, subjects were recruited from the outpatient and residential addiction treatment programs as well as medical and surgical units of Saint Mary’s Hospital and Methodist Hospital, which are affiliated with Mayo Clinic in Rochester, Minnesota. All alcohol-dependent subjects were of European descent, had a history of well-developed alcohol withdrawal including a subset of 39 subjects with a history of alcohol withdrawal-related seizures but no history of delirium tremens. Inclusion and exclusion criteria, enrollment procedures and evaluations are described elsewhere [1]. In brief, 190 male and female subjects, 18 years or older, meeting DSM-IV (American Psychiatric Association, 2000) criteria for alcohol dependence with a history of alcohol withdrawal with seizures or alcohol withdrawal without seizures were included in the study. Presence of seizures was identified as “at least one episode of witnessed loss of consciousness accompanied by tonic-clonic convulsions, which resulted in medical evaluation and/or occurred at a medical facility”. Only seizures occurring within 7 days after cessation of alcohol use were included. We excluded subjects with a history of psychotic or bipolar disorders; use of substances known to potentially cause seizures or delirium (e.g. barbiturates, benzodiazepines, opiates, hallucinogens, stimulants, cocaine, Phencyclidine (PCP), anticholinergics, etc.) at the time of the first episode of seizures or delirium; neurologic conditions with seizures and/or Electroencephalography (EEG) or imaging data indicating presence of the potentially epileptogenic focus prior to the first episode of seizures or delirium; and presence of somatic or metabolic conditions capable of provoking seizures and/or delirium at the time of the index episode (e.g. fever > 40°C, sodium < 120 or > 160 mEq/l, glucose < 60 mg%, magnesium < 1.0 mEq/l, calcium < 6.0 mEq/l, ammonia > 50 mg N/dl, BUN > 100 mg%, creatinine > 5.0 mg% or osmolality 350 mOsm/l). Medical records of potential study participants were reviewed for the presence of the inclusion and exclusion criteria, and those deemed eligible were invited to participate in the study. Subjects were interviewed by a board-certified addiction psychiatrist (VMK) and detailed information about the time line of the development of major elements of alcohol dependence was collected. For the purposes of this study, only data related to the presence or absence of seizure history as well as a history of alcohol consumption were included in the analyses. As population controls for the 190 alcohol-dependent subjects enrolled at Mayo Clinic, we used genomic DNA from 95 self-reported non-alcoholic subjects obtained from the Coriell Institute (www.coriell.org). The replication sample consisted of subjects recruited at the Ludwigs-Maximilians-University of Munich, Germany. The local ethical committee of the Ludwigs-Maximilians-University of Munich approved the study protocol; patients and controls were enrolled in the study after they gave written informed consent. Description of the study sample and evaluations have been described elsewhere [2,3]. In brief, 363 alcohol-dependent subjects with history of alcohol withdrawal, including 40 with a history of alcohol withdrawal-related seizures but no history of delirium tremens, were recruited from a ward for treatment of alcohol dependence. All patients were older than 18 years and met ICD10 and DSM-IV criteria for alcohol dependence. Characteristics of alcohol dependence were assessed with a structured interview, Semi-Structured Assessment for the Genetics of Alcoholism (SSAGA) [4,5]. These characteristics included age at onset of alcohol and/or nicotine dependence, average alcohol and nicotine consumption during the last week before admission, maximum quantity of alcohol ever consumed in 24 h, duration of alcohol dependence, lifetime and current history of withdrawal symptoms and delirium tremens and number of DSM-IV and ICD 10 alcohol dependence criteria endorsed. Daily alcohol intake data was quantified using the typical daily average alcohol consumption of the last 7 days before admission. Pure alcohol intake was computed in g/day. In addition, 435 non-alcoholic controls were recruited from the general population in Southern Germany. A comprehensive medical and psychiatric assessment including the SSAGA, as well as personality questionnaires (MMPI, NEO-FFI, TCI) and a short structured interview with a psychiatrist was carried out in all control persons together with routine laboratory screening. All persons with severe physical or psychiatric axis I or axis II disorders such as schizophrenia, depression, personality disorders and substance use disorders including alcohol dependence as well as those with any first-degree relative with a history of any axis I disorders (including alcohol dependence) were excluded.

**Statistics.** Tests of Hardy Weinberg Equilibrium were performed for each of the genotyped samples. The Mayo Clinic discovery sample was first used to test for association of alcohol dependence with the ENT1 647C (Ile216Thr) SNP using logistic regression analysis, which compared 190 alcohol-dependent subjects with 95 non-alcoholic controls. Results were confirmed by Fisher’s exact test as needed to account for low numbers of subjects with rare alleles. To define a more specific phenotype, which is more closely related to the phenotype associated with ENT1 in the mouse model, a subset of the alcohol-dependent cases that had a history of alcohol withdrawal seizures was identified. Cases with a history of delirium tremens were excluded from this more homogeneous case group. Logistic regression analysis was then used to assess association of the genotype with this phenotype of alcoholism with a history of withdrawal seizures, by comparing this more homogeneous subset of alcoholic subjects with the non-alcoholic controls. To test for association between the ENT1 variant and withdrawal seizures, an analysis comparing genotype frequencies between the group of alcohol dependent subjects with a history of withdrawal seizures and the group of alcohol dependent subjects with no history of withdrawal seizures was also performed. For consistency with the previous analysis, subjects with a history of delirium tremens excluded from this analysis. The same analyses were repeated using the replication (Munich) sample. Finally, analyses were performed using the combined set of subjects (Mayo and Munich). In these analyses, a covariate denoting the enrollment site was used to account for the two sources of subjects. All logistic regression analyses were performed in SAS (version 9.1; SAS Institute Inc., Cary, NC).

**References**

1. Karpyak VM, Biernacka JM, Weg MW, Stevens SR, Cunningham JM, et al. (2010) Interaction of SLC6A4 and DRD2 polymorphisms is associated with a history of delirium tremens. Addict Biol 15: 23-34.

2. Preuss UW, Zill P, Koller G, Bondy B, Hesselbrock V, et al. (2006) Ionotropic glutamate receptor gene GRIK3 SER310ALA functional polymorphism is related to delirium tremens in alcoholics. Pharmacogenomics J 6: 34-41.

3. Zill P, Preuss UW, Koller G, Bondy B, Soyka M (2008) Analysis of single nucleotide polymorphisms and haplotypes in the neuropeptide Y gene: no evidence for association with alcoholism in a German population sample. Alcohol Clin Exp Res 32: 430-434.

4. Bucholz KK, Cadoret R, Cloninger CR, Dinwiddie SH, Hesselbrock VM, et al. (1994) A new, semi-structured psychiatric interview for use in genetic linkage studies: a report on the reliability of the SSAGA. J Stud Alcohol 55: 149-158.

5. Hesselbrock M, Easton C, Bucholz KK, Schuckit M, Hesselbrock V (1999) A validity study of the SSAGA--a comparison with the SCAN. Addiction 94: 1361-1370.
