# Supplementary figures and images for: Functional Role of the Polymorphic 647 T/C Variant of ENT1 (SLC29A1) and Its Association with Alcohol Withdrawal Seizures
Source: PLoS One. 2011 Jan 24;6(1):e16331. doi: 10.1371/journal.pone.0016331 (PMC3026043; doi:10.1371/journal.pone.0016331)

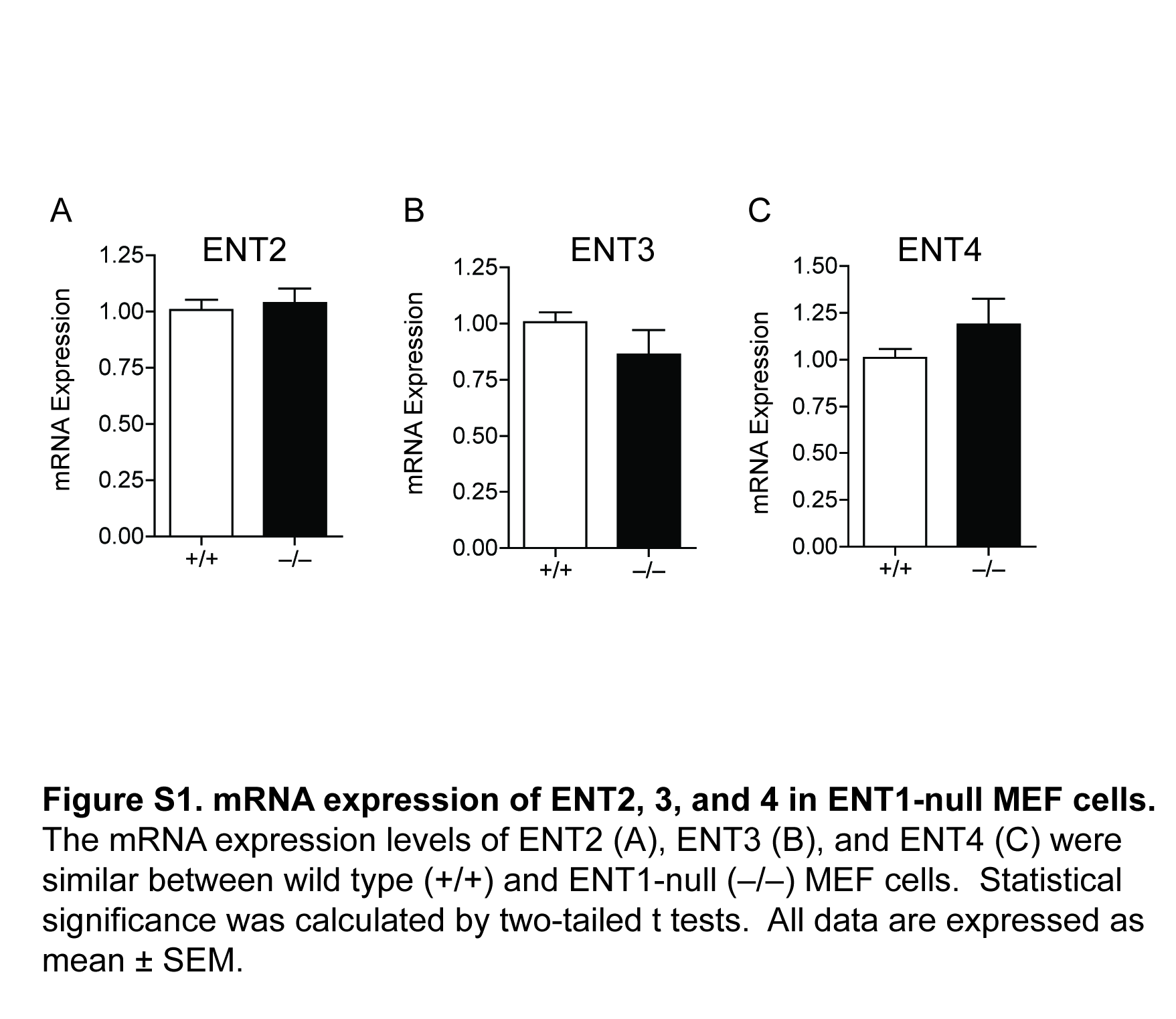

Supplement: Figure S1 — mRNA expression of ENT2, 3, and 4 in ENT1-null MEF cells. (TIF) [file pone.0016331.s002.tif]

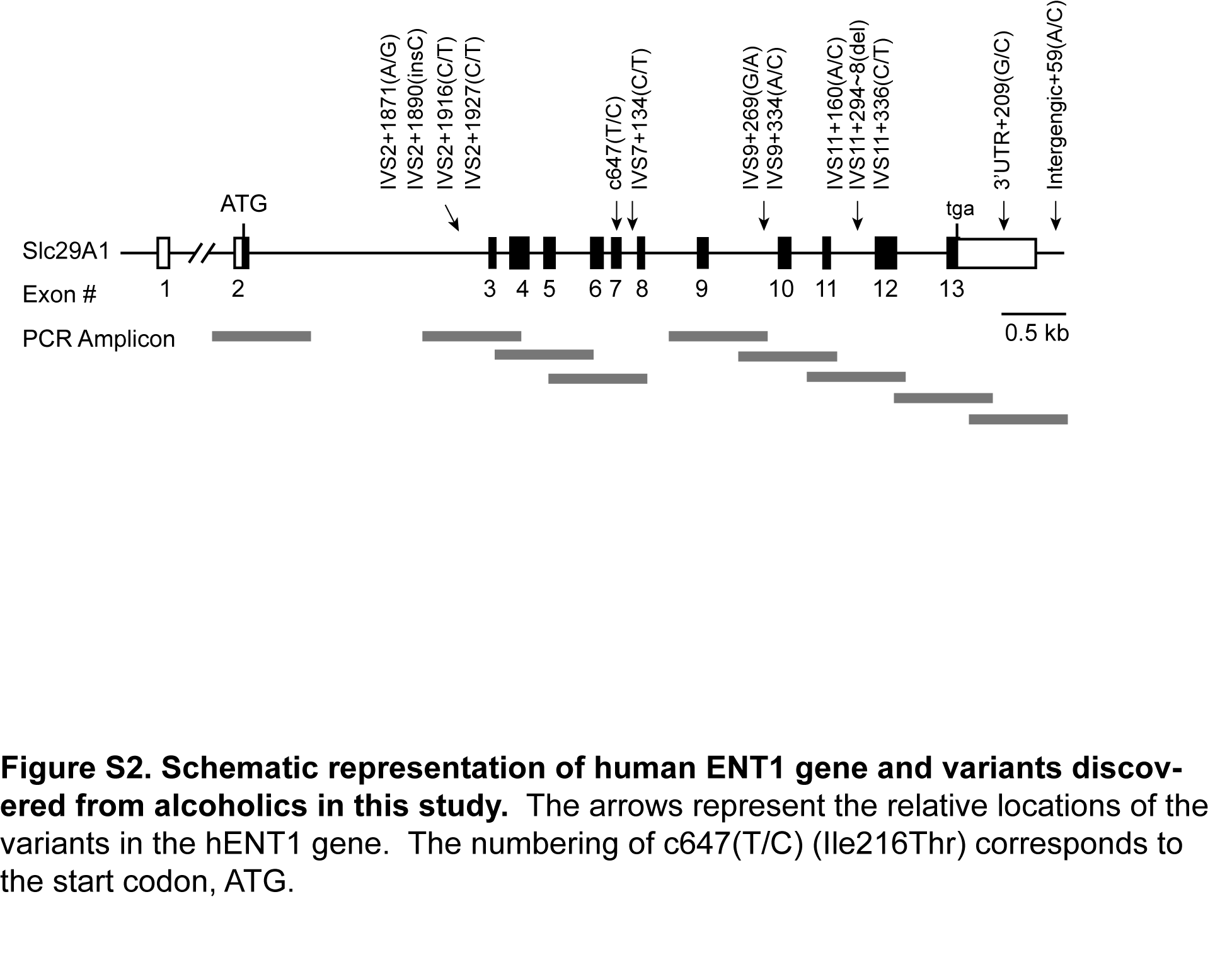

Supplement: Figure S2 — Schematic representation of human ENT1 gene and variants discovered from alcoholics in this study. (TIF) [file pone.0016331.s003.tif]
