# Supplementary material for: Functional Role of the Polymorphic 647 T/C Variant of ENT1 (SLC29A1) and Its Association with Alcohol Withdrawal Seizures
Source: PLoS One. 2011 Jan 24;6(1):e16331. doi: 10.1371/journal.pone.0016331 (PMC3026043; doi:10.1371/journal.pone.0016331)
Supplement: Table S1 — List of Primers for Genomic DNA Fragment Amplication for Resequencing. (DOC) [file pone.0016331.s004.doc]

| **Table S1.** List of Primers for Genomic DNA Fragment Amplication for Resequencing | | | |
| --- | --- | --- | --- |
|  |  |  |  |
| Primer | Sequence ( 5' to 3' ) | Fragment No. | PCR amplicon (bp) |
| S1 | TGGATAAAAATAGCGGTGGC | F1 | 595 |
| AS1 | CTGGAGAGCAACAGTGGAAAC |
| S2 | CCTTCCAGGGCTGATAACC | F2 | 654 |
| AS2 | GGAGTTGAGGTAGGTGAATAACAG |
| S3 | CCTTGGTCACTGCTGAACTG | F3 | 865 |
| AS3 | CGGACTTACTGGCAATAGCG |
| S4 | TTCCCCAGCATTTGGTGC | F4 | 737 |
| AS4 | CTGCCCCCATTTTATCATTG |
| S5 | GACTAAAGGCAGTGGGAAGC | F5 | 859 |
| AS5 | GGCTGAGGCAGGAGAATCAC |
| S6 | ACCAGGTCAAGCTGGTCTC | F6 | 706 |
| AS6 | ACAAATGGTCCCCTTACTA |
| S7 | TTCATCTCCTCTTCCAGAACG | F7 | 849 |
| AS7 | CCCTCCACTACAGTCACTATCCC |
| S8 | TGAAAGACAACCCCACCATAC | F8 | 553 |
| AS8 | GGAGAAACTCAAGCAAATGCC |
| S9 | ACTTCTGCTTTCCACGGCG | F9 | 747 |
| AS9 | GTCTTGGCTAACAGTCTTGGTG |
