# Supplementary material for: Functional Role of the Polymorphic 647 T/C Variant of ENT1 (SLC29A1) and Its Association with Alcohol Withdrawal Seizures
Source: PLoS One. 2011 Jan 24;6(1):e16331. doi: 10.1371/journal.pone.0016331 (PMC3026043; doi:10.1371/journal.pone.0016331)
Supplement: Table S2 — Genetic Variants of ENT1 (SLC39A1) in Alcohol-Dependent Subjects. (DOC) [file pone.0016331.s005.doc]

| **Table S2.** Genetic Variants of ENT1 (*SLC39A1*) in Alcohol-Dependent Subjects | | | | | | |
| --- | --- | --- | --- | --- | --- | --- |
|  |  |  |  |  |  |  |
| Chr. Position | dbSNP ID* | Variation | Amino Acid | Location | Minor Allele Frequency | |
|  |  |  |  |  | General ** | Alc (*n* = 50) |
| 44196950 | rs7750723 | A > G |  | IVS2+1871*** | N/A**** | 0.050 |
| 44196969 |  | insC |  | IVS2+1890 | N/A | 0.080 |
| 44196995 | rs324149 | C > T |  | IVS2+1916 | 0.240^†^ | 0.320 |
| 44197006 | rs41282660 | A > G |  | IVS2+1927 | N/A | 0.110 |
| **44198362** | **rs45573936** | **T > C** | **Ile216Thr** | **c647** | **0.021^†^** | **0.050** |
| 44198536 | rs41282662 | C > T |  | IVS7+134 | N/A | 0.010 |
| 44199467 |  | G > A |  | IVS9+269 | N/A | 0.010 |
| 44199532 |  | A > C |  | IVS9+334 | N/A | 0.010 |
| 44200325 | rs1128930 | A > C |  | IVS11+160 | N/A | 0.390 |
| 44200459 |  | delCCTGA |  | IVS11+294-298 | N/A | 0.010 |
| 44200501 |  | C > T |  | IVS11+336 | N/A | 0.010 |
| 44201474 |  | G > C |  | 3'UTR+209 | N/A | 0.010 |
| 44201947 |  | A > C |  | Intergenic+59 | N/A | 0.320 |
| *dbSNP ID, identification numbers of single nucleotide polymorphisms published on the website of the | | | | | | |
| National Center for Biotechnology Information (NCBI) | | | |  |  |  |
| **Minor Allele Frequency (MAF) are from Osato et al. (2003). | | | |  |  |  |
| ***IVS, intronic regions; 3’UTR, 3’-untranslated region. | | | |  |  |  |
| ****N/A, Not available. | |  |  |  |  |  |
