# Supplementary material for: Functional Role of the Polymorphic 647 T/C Variant of ENT1 (SLC29A1) and Its Association with Alcohol Withdrawal Seizures
Source: PLoS One. 2011 Jan 24;6(1):e16331. doi: 10.1371/journal.pone.0016331 (PMC3026043; doi:10.1371/journal.pone.0016331)
Supplement: Table S3 — Summary of Clinical and Demographical Information of Subjects. (DOC) [file pone.0016331.s006.doc]

| **Table S3.** Summary of Clinical and Demographical Information of Subjects | | | | | | |
| --- | --- | --- | --- | --- | --- | --- |
|  |  |  |  |  |  |  |
| Recruiting site | Mayo | | | Munich | | |
| Group | Non-Alc | Alc-All | Alc WS***** | Non Alc | Alc-All | Alc WS |
|  | (*n* = 95) | (*n* = 190) | (*n* = 39) | (*n* = 435 ) | (*n* = 363 ) | (*n* = 41 ) |
| Male (n(%)) | 47 (49.5%) | 137 (72.1%) | 31 (79.5%) | 224 (52%) | 276 (77%) | 34 (83%) |
| Age (mean ± SD) | 37.4 ± 16.3 | 52.0 ± 9.6 | 48.4 ± 10.5 | 42.8 ± 16.1 | 43.7 ± 9.9 | 43.3 ± 10.6 |
| Alcohol consumption (drinks/day)* | -- | 16.9 ± 9.4 | 19.0 ± 10.9 | -- | 20.4 ± 12.5 | 23.2 ± 16.0 |
| Maximum tolerance (drinks)** | -- | 24.2 ± 13.2 | 28.8 ± 17.0 | -- | 37.6 ± 24.8 | 43.1 ± 23.3 |
| Age regular use started (yr)*** | -- | 19.2 ± 5.5 | 17.7 ± 3.7 | -- | 21.4 ± 8.2 | 22.1 ± 8.5 |
| Length of drinks (yr)**** | -- | 29.2 ± 9.6 | 27.7 ± 9.7 | -- | 22.2 ± 10.2 | 21.2 ± 9.3 |
| *Average consumption per drinking day and drinks indicate a "standard drink" as defined by NIAAA (http://www.niaaa.nih.gov). | | | | | | |
| **Lifetime maximum number of drinks consumed per 24 hour period, | | |  |  |  |  |
| ***Drinking at lease once per month for at least 6 month in a row, | | |  |  |  |  |
| ****Years from age regular drinking started to age at evaluation, | | |  |  |  |  |
| *****Alc WS denotes that subgroup of alcohol-dependent subjects (Alc-All) with alcohol withdrawal seizures. | | | | | |  |
